# Improvement of Biosynthetic Ansamitocin P-3 Production Based on Oxygen-Vector Screening and Metabonomics Analysis

**DOI:** 10.1155/2022/3564185

**Published:** 2022-06-02

**Authors:** Xiaolin Zhu, Kaiyao Hou, Peiyang Zheng, Wenya Zhong, Jing Guo, Xiyue Zhao, Tingting Hong, Zhiqiang Cai

**Affiliations:** ^1^Laboratory of Applied Microbiology and Enzyme Engineering, School of Pharmacy, Changzhou University, Changzhou 213164, Jiangsu, China; ^2^Wuxi Big Bridge Academy, Wuxi 214115, Jiangsu, China

## Abstract

A novel approach involving exogenous oxygen vectors was developed for improving the production of biosynthetic Ansamitocin P-3 (AP-3). Four types of oxygen vectors including soybean oil, n-dodecane, n-hexadecane, and Tween-80 were applied to explore the effect of exogenous oxygen vectors on AP-3 yield. It was observed that soybean oil exhibited a better ability for promoting AP-3 generation than the other three oxygen vectors. Based on the results of the single-factor experiment, response surface methodology was employed to obtain the optimal soybean oil addition method. The optimum soybean oil concentration was 0.52%, and the addition time was 50 h. Under this condition, the yield of AP-3 reached 106.04 mg/L, which was 49.48% higher than that of the control group without adding oxygen vectors. To further investigate the influence of dissolved oxygen on precious orange tufts actinomycetes variety *A*. *pretiosum* strain metabolism and AP-3 yield, metabolomics analysis was carried out by detecting strain intermediate metabolites at various stages under different dissolved oxygen levels. Moreover, differential metabolite screening and metabolic pathway enrichment analysis were combined to exploit the effect mechanism of soybean oil on AP-3 production. Results suggested that primary metabolic levels of the TCA cycle and amino acid metabolism increased with the increase in dissolved oxygen level, which was beneficial to the life activities of bacteria and the synthesis of secondary metabolic precursors, thus increasing the production of AP-3.

## 1. Introduction

Maytansine has attracted tremendous attention due to its significant antitumor effect [1, 2]. Nevertheless, it is difficult to synthesize maytansine due to its complex chemical structure, and the chemical synthesis method is limited to laboratory research. Moreover, the plants containing lignin alkaloids exist in tropical regions, and the maytansine content in plants is low. Therefore, it is necessary to look for compounds with similar biological activity to maytansine. Biogenic ansamitocin is considered a suitable alternative since it possesses similar antitumor properties as maytansine. Higashide et al. first isolated ansamitocin from *Nocardia sp*., and this group of new components presented strong antitumor characteristics [3]. Different types of side chains can be modified to the Ansamitocin C-3 hydroxyl group to obtain amitocin homologues, including Ansamitocin P-0, Ansamitocin P-1, Ansamitocin P-2, Ansamitocin P-3, Ansamitocin P-3′, and Ansamitocin P-4. Among these homologues, Ansamitocin P-3 (AP-3) containing isobutyryl side chain shows prominent antitumor activity and has attracted increasing attention in secondary metabolite research [4–8]. The antibody-drug coupling (ADC) agent T-DM1 has achieved desirable results in the treatment of breast cancer [9–13]. In 2019, trastuzumab emtansine, the first ADC drug for early breast cancer treatment in China, was approved in the United States at an expensive price of about ¥ 20,000. High production cost of AP-3 due to low production efficiency is one of the main problems restricting its wide application in the pharmaceutical field.

The commonly used strains for biosynthesizing AP-3 include the precious orange tufts actinomycetes variety *A*. *pretiosum* and the miracle fasciculus actinomycetes *A*. *mirum.* Nowadays, increasing the yield of AP-3 is still a hot research area because of the low production of AP-3 fermentation. To promote the biosynthesis of AP-3, researchers have adopted various strategies including mutant screening, medium optimization, and genetic modification [14–18]. It is of great significance to the industrial application of AP-3. Desirable strains are the basis of fermentation, and the mutagenesis breeding method is usually adopted to obtain the mutant strains with a high secondary metabolite yield. In addition, the optimal medium has an important effect on increasing AP-3 production. As the basic elements for promoting strain growth and proliferation, the medium components can provide energy for strain metabolism and regulate the primary and secondary metabolism of strains. For instance, glycerol and glucose synergistically promoted the production of AP-3. As a delayed carbon source, glycerol could provide nutrients for strain growth in the late fermentation period [19]. Since fructose offered precursors for AP-3 biosynthesis, the yield of AP-3 reached 144 mg/L in the optimized medium with fructose as the main carbon source [20].

In the process of industrial fermentation of aerobic microorganisms, the level of dissolved oxygen in the fermentation broth is one of the key factors affecting the product yield. The formation of mycelium is able to increase the fermentation broth viscosity, resulting in a rapid decrease in the dissolved oxygen level. Increasing the rotational speed and utilizing pure oxygen can solve the problem of oxygen transfer restriction. Nevertheless, the shear force caused by high stirring and ventilation has adverse effects on mycelium morphology and product yield. In addition, increased ventilation can lead to higher energy consumption. It is necessary to develop an effective way to improve the oxygen transfer rate during aerobic microbial fermentation. The oxygen transfer rate from the gas phase to microbial cells can be improved by adding certain organic liquids to the fermentation broth. These liquids are commonly referred to as oxygen vectors and include n-alkanes, n-fluorocarbons, esters, fatty alcohols, and oils [21–26]. Since the solubility of oxygen in these organic liquids is several times or dozens of times higher than that in the medium, these oxygen vectors are widely used in the field of biochemical engineering [26]. Compared with traditional fermentation systems, oxygen vectors can enhance the oxygen transfer rate without additional energy supply. Moreover, low-dose oxygen vectors have nontoxic effects on microorganisms and may become additional substrates. Therefore, oxygen-vector-based fermentation systems have attracted extensive attention. For instance, Meyer et al. adopted bioinert perfluorocarbons to improve oxygen transfer in 96-well plates [27]. It was found that PFCs were able to influence the solubility and transport of oxygen without interfering with the medium composition. As a common oxygen vector, n-alkanes play an important role in the fermentation process of polysaccharides and antibiotics. This type of oxygen vector can quickly transfer oxygen to the fermentation liquid and improve the oxygen uptake rate of bacteria. An additional 4% n-hexadecane was added to the culture medium of *S*. *cerevisiae* [28]. The results indicated that n-hexadecane could increase glucose consumption and reduce ethanol accumulation, facilitating *S*. *cerevisiae* growth and adenosine-methionine synthesis. To increase the available dissolved oxygen, Ciobanu et al. utilized 2% n-dodecane as the oxygen vector [29]. The production of *β*-galactosidase could be enhanced. To the best of our knowledge, few studies have specifically focused on investigating the effect of oxygen vectors on the production of biosynthetic AP-3.

Metabolomics is an emerging field of systems biology that aims to comprehensively analyze metabolites of cells at certain times and under certain environmental conditions [30]. It has also become a popular tool in biotechnology nowadays. Metabolomics is the systematic study of all metabolites and their concentrations that are affected by pathological and physiological changes. Metabolic changes present the complicated interaction between gene expression, enzyme activity, and metabolic reactions. Metabolomics is mainly applied for studying structurally different and physicochemically different molecules, including lipids, sugars, ions, metabolic intermediates, and biochemical reaction products, as well as the building blocks of proteins, nucleic acids, and cell membranes. This results in the technical barriers in terms of dynamic range and comparability. Technological developments, along with new data analysis methods, have played a key role in the field of metabolomics. Metabolomics has been applied to various aspects of microbiology, such as phenotypic classification, fermentation processes, and metabolic pathways [31–34]. Metabolite mapping facilitates the discovery of biological and chemical diversity from analytical data, further promoting the understanding of species, phenotypes, and functional genomics. Therefore, metabolomics is considered an important topic in microbial taxonomy and physiological research. Smedsgaard et al. used metabolite profiles to analyze the information related to *Penicillium* species. Phenotypes were effectively identified and classified by integrating efficient analytical methods, data processing techniques, and intelligent screening [35]. Dalluge et al. utilized liquid chromatography-electrospray tandem mass spectrometry (MS) to rapidly quantify underived amino acids. The metabolism of 20 amino acids was able to be monitored during microbial fermentation [36]. Although detailed metabolic networks have been established for some model strains, information about the relationships between primary and secondary metabolites and the transition patterns of entire microorganism metabolites is unclear. It is necessary to develop a more comprehensive method based on metabolomics for analyzing these secondary metabolite production pathways. Untargeted metabolomics was used to analyze the secondary Streptococcus A3(2) cultured in two different media, and the production of specific secondary metabolites was analyzed by the metabolic pathway, which could be applied to further optimize the production of schizopeptide [37].

**Figure 1:**
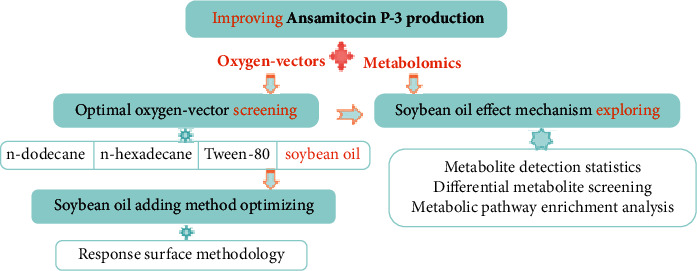
Schematic drawing of improving AP-3 production based on oxygen-vector screening and metabonomics analysis.

To take advantages of both oxygen vectors and metabolomics, herein, the oxygen utilization efficiency of AP-3 production strains was improved by utilizing an oxygen vector and the effect mechanism of dissolved oxygen on AP-3 biosynthesis was investigated via metabonomics analysis (Figure 1). The influence of different types of oxygen vectors on AP-3 yield was explored by using the single-factor experiment. To further improve AP-3 production and offer a reference for other antibiotic fermentation methods with high production, response surface methodology (RSM) was applied for choosing the optimal oxygen-vector addition method. The effect of dissolved oxygen on cell metabolism in the biosynthesis of AP-3 was analyzed by untargeted metabonomics. The comparative analysis of the relationship between oxygen vectors and cells facilitated the investigation of the AP-3 yield enhancement mechanism of the oxygen vector during the fermentation process, providing a metabolic regulation approach to improve AP-3 yield. This work opens the way to applying oxygen vectors in the industrial production of AP-3.

## 2. Materials and Methods

### 2.1. Strain and Medium

The precious orange tufts actinomycetes variety *A*. *pretiosum* strain B24-13 was obtained by UV-induced mutation. Before the fermentation process, the basal medium was prepared. The basal medium composition was as follows: saccharose 2.5 g/L, glycerin 1.5 g/L, corn plasm 2.5 g/L, isobutanol 0.2 g/L, calcium carbonate 0.7 g/L, magnesium sulfate heptahydrate 0.05 g/L, L-valine 0.05 g/L, and iron sulfate heptahydrate 0.001 g/L. The spores were germinated, and the cells were grown at 28°C in an incubator shaker, at 220 rpm, for 7 days.

### 2.2. Screening Optimal Oxygen Vectors

Four types of oxygen vectors including soybean oil, n-hexadecane, n-dodecane, and Tween-80 were investigated in this experiment. Soybean oil and Tween-80 were directly sterilized, and n-hexadecane and n-dodecane were filtered for sterilization. A shaker fermentation medium without oxygen vectors was used as the control. The initial addition concentration was 1% (v/v), and each group performed 3 parallel experiments. After fermentation, the optimal oxygen vector promoting AP-3 biosynthesis was selected by detecting the dry weight of bacteria and AP-3 production in each shaker. After screening the optimal oxygen vector, oxygen-vector concentrations of 0%, 0.5%, 1.0%, 2.0%, and 4.0% (v/v) were examined and 3 parallel experiments were set up in each group. Furthermore, the oxygen vector with the optimal concentration was designed to be added at 0 h, 24 h, 48 h, 72 h, 96 h, and 120 h after inoculation and culture. After fermentation, the optimized addition time of the oxygen vector was explored via the detection of dry weight and AP-3 yield of bacteria. Based on the results of the single-factor optimization experiment, RSM was applied to optimize the addition process of the oxygen vector by choosing AP-3 production as the testing parameter. Design-Expert software was utilized to carry out experimental design and data analysis of the optimal oxygen-vector addition program. The response surface model was further verified by investigating the reliability and accuracy of the optimal oxygen-vector additive process optimized by the model.

### 2.3. Measurement and Analysis Methods

The AP-3 amount accumulated during fermentation was analyzed by the HPLC approach (Agilent 1260 system utilizing a Shim-pack GIST C_18_ column 250 × 4.5 mm, 5 *μ*m) with 70% methanol as the mobile phase. The mobile phase flow rate was 0.8 mL/min, and the analysis temperature was 25°C. The HPLC system was equipped with a PDA detector. AP-3 was detected at 254 nm. Moreover, metabolite analysis was performed by using HPLC-MS (ACQUITY UPLC HSS T3 column 100 × 2.1 mm, 1.8 *μ*m, mobile phase: (A) 0.1% formic acid solution and (B) 0.1% formic acid-acetonitrile solution, at a flow rate of 0.35 mL/min). For sample pretreatment, about 1 mL of the culture was pumped into precooled methanol-water solution (V : V = 4 : 1) with a rapid sampler within 0.2 s, and then, 200 *μ*L of precooled chloroform was added. The mixture was crushed by ultrasonic method in an ice bath and transferred to the EP tube. Subsequently, 20 *μ*L of internal standard (L-2-chlorophenylalanine) was added, and the sample was put into vials and dried. After being dissolved in 200 *μ*L methanol-water solution, the sample was stored at −20°C for 2 h and then centrifuged. The supernatant of 150 *μ*L was filtered through an organic filter and transferred to a sample vial, and metabolites were detected by LC-MS. For MS assay, an electrospray ion source was chosen and MS was performed in the positive or negative ionization mode. The experiments were repeated three times, and the average value of measured parameters was utilized in calculations. For dry cell weight detection, the centrifugated bacteria were washed twice with distilled water and then dried in an oven at 60°C and the mass gain was measured.

## 3. Results and Discussion

### 3.1. Effect of Oxygen Vector on AP-3 Production

**Figure 2:**
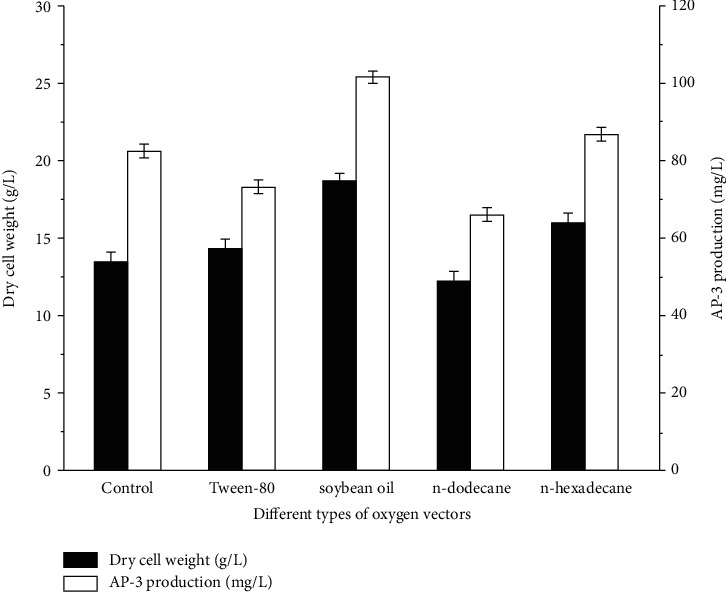
Effect of oxygen-vector types on AP-3 biosynthesis.

Four types of oxygen vectors including soybean oil, n-dodecane, n-hexadecane, and Tween-80 were investigated to improve AP-3 production. As shown in Figure 2, oxygen vectors except n-dodecane can promote strain growth. AP-3 production decreased in the order of soybean oil > n-hexadecane > Tween-80. The most efficient fermentation for AP-3 production (101.66 mg/L) was that by choosing soybean oil as the oxygen vector. In addition, soybean oil could be used as a carbon source in the fermentation medium, facilitating strain B24-13 differentiation, sporulation, and secretion of secondary metabolites. Nevertheless, AP-3 yield of the Tween-80 group reduced when compared to that of the control group. It might be attributed to the fact that Tween-80 could be applied as a nutritional resource for microbial growth. Since the biosynthesis of AP-3 was influenced by various factors, Tween-80 was not suitable for promoting AP-3 generation. Therefore, soybean oil was selected as the optimum oxygen vector for in-depth research.

The impact of different concentrations of soybean oil on AP-3 production was investigated in this research. It was observed that AP-3 production could reach 100.33 mg/L when adding only 0.5% soybean oil. However, AP-3 production decreased slightly with soybean oil concentration increasing from 0.5% to 4%. It was found that 4% soybean oil was unfavorable for producing AP-3 and could inhibit cell growth. This phenomenon might be attributed to the fact that more water loss occurred in the fermentation medium under the condition of less liquid content and higher dissolved oxygen. Therefore, 0.5% was chosen as the optimum soybean oil concentration. Furthermore, the effect of the soybean oil addition time on AP-3 biosynthesis was explored. The microbial growth speed and AP-3 production were enhanced obviously when soybean oil was added after 48 h inoculation. Since large amounts of lactic acid and ethanol existed in the fermentation medium, the strain was in a state of oxygen starvation. The addition of soybean oil could effectively solve the dissolved oxygen limitation that occurred in the fermentation system of strain B24-13.

### 3.2. Response Surface Methodology

**Figure 3:**
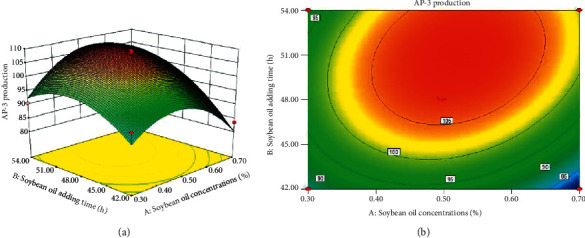
(a) Response surface and (b) contour map of the effect of soybean oil concentration and addition time on AP-3 production.

Based on single-factor tests, the RSM was used to optimize the addition strategy of soybean oil. As presented in Table 1, the factor levels were coded as −1 and +1 and central points were coded as zero values. Two axial points on the axis of each design variable at a distance of ±*α* from the design centre were chosen as ±*α*. A total of 13 experiments were conducted (Table 2). The fitting equation of AP-3 production and the test variables (A: soybean oil concentration and B: soybean oil addition time) was *Y* = −384.549 + 98.065*A* + 18.387*B* + 3.25AB−252.563*A*^2^−0.197*B*^2^. Variance analysis and significance test were applied for exploring regression effects. The analysis of variance is given in Table 3. The model F-value of 21.37 indicated that the model was significant. As we know, the smaller the magnitude of the *P* value, the more significant is the corresponding coefficient. And, a *P* value less than 0.05 demonstrates that the model terms are significant. Therefore, the Fisher F-test with a low probability value (model *P* value (Prob > *F*) = 0.0004) also suggested a high significance for the regression model and verified the adequacy of the quadratic model. The regression coefficient of this fitting result was 0.94 (*R*^2^), indicating that the fitting data were in good correlation with the monitoring data. As seen in Figure 3, the optimum soybean oil concentration was 0.52% and the addition time was 50 h. The theoretical maximum yield of AP-3 (108.95 mg/L) could be calculated by substituting levels of the factors into the regression equation. Moreover, the maximum AP-3 production obtained experimentally under the optimized condition was 106.04 mg/L, an improvement of 49.48% compared with that of the control group without adding oxygen vectors.

### 3.3. Fermentation Curve

**Figure 4:**
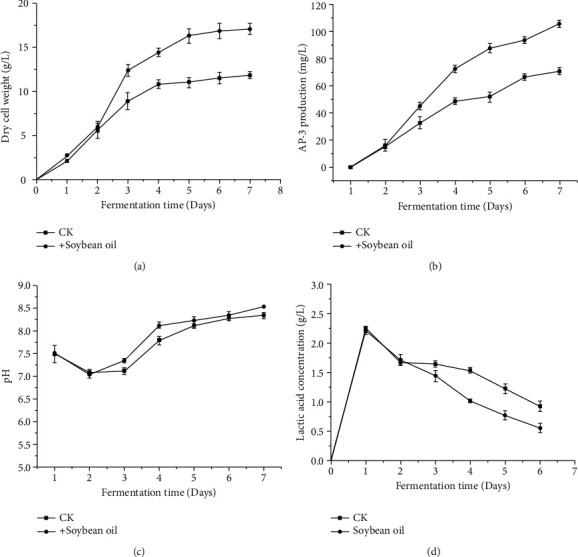
(a) DCW, (b) AP-3 production, (c) pH, and (d) lactic acid content before and after adding soybean oil.

After 48 h from the start of fermentation, AP-3 production increased obviously and the dry cell weight increased rapidly because of the addition of soybean oil (Figures 4(a) and 4(b)). The results indicated that the level of dissolved oxygen directly influenced the growth state of strain B24-13, further affecting the synthesis efficiency of metabolites. During the fermentation process of microorganisms, higher oxygen supply promoted the aerobic metabolism of strain B24-13, facilitating AP-3 biosynthesis and strain growth. When strain B24-13 was in the stable phase, the secondary metabolism took place. And, AP-3 production of the experimental group was 106.04 mg/L, 49.48% higher than that of the control group (Figure 4(a)). Under the condition of insufficient dissolved oxygen, pyruvate anaerobic respiration could produce lactic acid. It was observed that the pH value of the control group was lower than that of the experimental group after 48 h of fermentation, suggesting that the utilization of soybean oil was able to enhance the oxygen uptake ability of the strains (Figure 4(c)). To further explore the underlying causes of improving AP-3 production via soybean oil, the metabolites from fermentation were used for metabonomics studies.

### 3.4. Metabolite Detection Statistics and Differential Metabolite Screening

All metabolites obtained were analyzed qualitatively and quantitatively. Positive and negative models were developed for screening 3765 and 1709 metabolites, respectively. To obtain the pathway information with high genetic similarity with strain B24-13, the identified metabolites were matched with the KEGG database. The related metabolites were screened, including (1) amino acids such as tyrosine (Tyr), proline (Pro), and arginine (Arg); (2) intermediate metabolites in the glycolysis pathway such as glucose-6-phosphate (G6P), 3-phosphoglyceride (3PGA), and pyruvic acid (Pyr); (3) organic acid in the tricarboxylic acid (TCA) cycle such as citric acid, ketoglutaric acid (Akg), and oxaloacetic acid (Oaa); and (4) precursor substances in the Ap-3 biosynthesis process such as UDP-glucose (UDPG) and methoxymalonyl-ACP. These differential metabolites almost cover the AP-3 biosynthesis pathway, facilitating the investigation of intracellular states of strain B24-13 at different dissolved oxygen levels for producing AP-3. To further explore the differences between metabolites, more work still needs to be focused on analyzing the enrichment pathway of differential metabolites.

### 3.5. Enrichment Analysis of Metabolic Pathways

**Figure 5:**
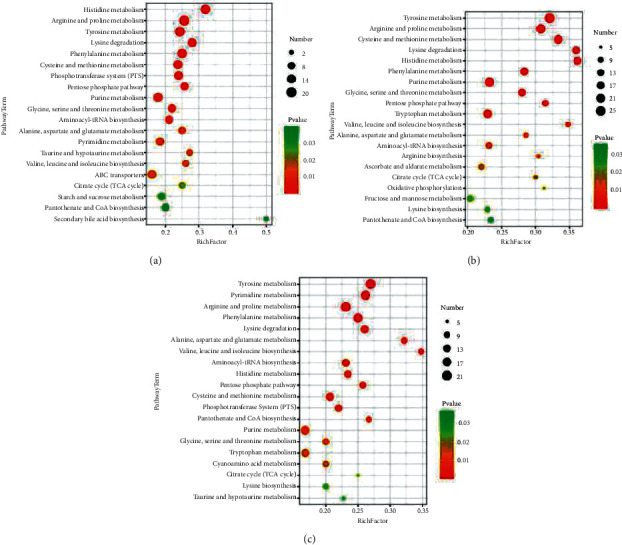
KEGG pathway enrichment analysis of differential metabolites on (a) day 3, (b) day 5, (c) and day 7.

KEGG pathway enrichment analysis (*P* value ≤ 0.05) of strain B24-13 differential metabolites in different fermentation stages was carried out (Figure 5). The results indicated that the addition of soybean oil could obviously affect the amino acid-related metabolic pathways. And, metabolite enrichment of tyrosine metabolism, lysine degradation, and arginine and proline metabolism was the most significant. The pentose phosphate pathway (PPP) and the tricarboxylic acid cycle (TCA) ran through the entire fermentation stage of strain B24-13. When the strain entered the stable phase from the logarithmic phase, we found that the time of producing enzymes coincided with the time when a series of amino acid metabolic pathways were significantly enriched. This suggested that the metabolism of amino acids into tissue proteins and their metabolites could promote AP-3 biosynthesis. The metabolic enrichment changes of alanine, aspartic acid, and glutamic acid metabolism and valine, leucine, and isoleucine biosynthesis were significant. Therefore, the application of the soybean oil oxygen vector facilitated various amino acid metabolisms, further motivating AP-3 production.

### 3.6. Analysis of Significant Metabolic Pathways of Differential Metabolites

**Figure 6:**
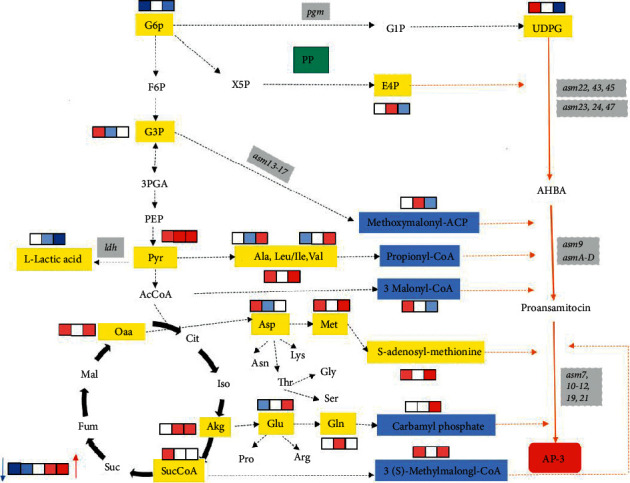
Changes in the AP-3 biosynthetic pathway affected by soybean oil.

To further explore the effect of dissolved oxygen on the metabolism of strains and the improvement of AP-3 production, changes in the intracellular metabolite content in the presence of oxygen vector were explored by analyzing central carbon metabolism, amino acid metabolism, and AP-3 synthesis pathway. As seen in Figure 6, the pentose phosphate pathway (PP) and glycolysis pathway (EMP) were downregulated, indicating that the added soybean oil could increase the oxygen uptake rate of strain B24-13 and consume primary metabolic intermediates for aerobic metabolism. Nevertheless, the intermediate metabolites of the tricarboxylic acid (TCA) cycle were upregulated, suggesting that soybean oil was able to enhance the TCA cycle metabolism, further providing precursors for biosynthesizing AP-3. This result coincided with the utilization of oxygen vectors for improving *Streptomyces albus* PD-1 metabolism to synthesize *β*-phospholipase [38]. The intermediate metabolites produced by central carbon metabolism including erythritol-4-phosphate (E4P), phosphoenolpyruvic acid, propionyl-CoA, methylmalonyl-ACP, and methylmalonyl-CoA were precursors of AP-3 biosynthesis.

The relative content of pyruvate (Pyr) in the EMP pathway was significantly upregulated. On the one hand, Pyr came into contact with amino acid metabolic pathways or lactic acid. On the other hand, Pyr went into TCA cycle metabolism through acetyl-CoA. Therefore, changes in pyruvate concentration might reveal changes in dissolved oxygen levels in the fermentation environment, which played an important role in metabolic regulation. Moreover, the intermediate products of the TCA cycle including *α*-ketoglutarate (Akg), succinic acid-CoA, and oxaloacetic acid (Oaa) were significantly upregulated during the stationary phase of fermentation. These metabolites could be converted to precursors for AP-3 biosynthesis. These results highlighted the critical influence of oxygen vectors on the efficiency of biological processes and the metabolic behavior of host cells, which directly affected the production of metabolites.

As shown in Figure 6, the relative contents of amino acids were significantly upregulated in the presence of soybean oil, indicating that the addition of soybean oil increased the dissolved oxygen level of fermentation, further promoted the bacteria, and synthesized a large number of amino acids for AP-3 biosynthesis. The aspartic acid (Asp) and the glutamate (Glu) were used as ammonia donors for, respectively, biosynthesizing methionine (Met) and glutamine (Gln), further providing compounds for producing carbamoyl phosphate, S-adenosine-methionine, and other AP-3 precursors. Pyruvate-derived alanine (Ala), valerian (Val), leucine (Leu), and isoleucine (Ile) were the precursors of methylmalonyl-CoA biosynthesis. The relative contents of these amino acids were significantly upregulated, which was beneficial to AP-3 production.

UDP-glucose (UDPD) was the important precursor of AP-3 biosynthesis, and it could be converted to glucose 1-phosphate (G1P) by using glucose 6-phosphate mutase (PGM). Subsequently, G1P was converted to UDPD and produced 3-amino-5-hydroxyl-benzoic acid (AHBA) precursor through the amino-shikimate route. It was observed that the phosphorylation level was significantly increased and the relative content of UDPD was significantly upregulated when the dissolved oxygen level was high. During the AP-3 rapid synthesis period, the UDPD concentration decreased rapidly because of synthesizing AP-3. This was similar to the effect of adding oxygen vectors on metabolic flux distribution in *Bacillus subtilis* NX-2 fermentation process [39]. As a precursor of AP-3 biosynthesis, S-adenosyl-methionine could participate in the extension and modification process of the polyketone carbon chain. When the dissolved oxygen level was high, S-adenosyl-methionine concentration upregulated during the stationary phase, facilitating the increase in AP-3 yield. In conclusion, the addition of soybean oil increased the dissolved oxygen level in the fermentation process, which was beneficial to the energy metabolism of strains, further improving AP-3 production.

### 3.7. VGB Gene Expression in the A. *pretiosum* Strain B24-13

To further improve the yield of AP-3, the genetic performance of strain B24-13 was improved via genome shuffling technology. In this work, three strategies including PEG-mediated protoplast transformation, electrotransformation, and conjugation transformation were applied to introduce the exogenous VGB gene into the A. *pretiosum* strain B24-13. By promoting oxygen delivery, oxygen utilization, cell growth, respiration, metabolism, and AP-3 biosynthesis yield might be improved. The recombinant plasmid pET28a-VGB was transformed into *E*. *coli* BL 21, and the expression level of VHb protein was analyzed by using SDS-PAGE. Unfortunately, no transformant was obtained when recombinant plasmid pIJ86-VGB was constructed for protoplast transformation and electrotransformation. The conditions of the transformant need to be further explored. In addition, the recombinant plasmid PIB139-VGB was constructed and the conjugation transformation condition was investigated. The optimal medium was determined to be the MS medium. The concentration ratio of the donor and recipient was 1 : 1, and the best coverage period of the antibiotic solution was 18 h. More effort needs to be put into screening positive zygotes.

## 4. Conclusion

Four types of oxygen vectors including soybean oil, Tween-80, n-dodecane, and n-hexadecane were utilized to improve AP-3 yield. Results suggested that soybean oil was the optimum oxygen vector. As the concentration of the oxygen vector was an important factor in the AP-3 biosynthesis process, the impact of different concentrations of soybean oil on AP-3 production was investigated in this research. The optimized addition concentration of soybean oil was 0.5%, and the addition time was 48 h. Furthermore, the optimal addition strategy of soybean oil was explored by using RSM based on the single-factor optimization experiment. We found that the optimum addition amount of soybean oil was 0.52% and the addition time was 50 h. Under this condition, the maximum AP-3 production was 106.04 mg/L. In comparison with the control group without adding soybean oil, an improvement of 49.48% was observed. Metabonomics analysis based on LC-MS was carried out to investigate the intermediate metabolites of the strain at various stages at different dissolved oxygen levels. Positive and negative models were developed for quantitatively screening 3765 and 1709 metabolites, respectively. The metabolites associated with precious orange tufts actinomycetes include amino acids, EMP intermediate metabolites, organic acids in the TCA cycle, and precursors of the AP-3 biosynthesis pathway. The enrichment results of metabolic pathways and the fermentation metabolism curve showed that the TCA cycle and amino acid metabolism increased with the increase in fermentation dissolved oxygen level, which promoted the life activities of bacteria and improved metabolic flux of secondary metabolism. In addition, genetic engineering was investigated to obtain oxygen-carrying genes to improve the oxygen utilization rate of strains. Further work needs to be focused on screening desirable zygotes.

## Figures and Tables

**Table 1:** Test factors and levels.

Level	Factors	
	A: soybean oilconcentration (%)	B: soybean oiladdition time (h)
−*α*	0.22	39.51
−1	0.3	42
0	0.5	48
1	0.7	54
*α*	0.78	56.49

**Table 2:** Response surface experiment approach and results.

No.	Factor 1	Factor 2	Response
	A: soybean oil concentration (%)	A: soybean oil addition time (h)	AP-3 production (mg/L)
1	0.7	54	98.3
2	0.5	48	109.4
3	0.78	48	87.3
4	0.3	54	90.5
5	0.5	39.51	79.2
6	0.3	42	91.3
7	0.5	48	107.1
8	0.22	48	85.6
9	0.5	56.49	105.7
10	0.5	48	104.5
11	0.5	48	108.6
12	0.7	42	83.5
13	0.5	48	107.3

**Table 3:** Variance analysis of regression model.

Variation source	Quadratic sum	Degree of freedom	Mean square	F-value	*P* value (prob > *F*)
Model	1339.6	5	267.92	21.37	0.0004^*∗*^
A: soybean oil concentrations (%)	0.72	1	0.72	0.058	0.8172
A: soybean oil addition time (h)	331.23	1	331.23	26.42	0.0013^*∗*^
AB	60.84	1	60.84	4.85	0.0635
*A* ^2^	709.99	1	709.99	56.62	0.0001^*∗*^
*B* ^2^	350.93	1	350.93	27.99	0.0011^*∗*^
Residual error	87.77	7	12.54		
Lack of fit	73.82	3	24.61	7.06	0.0448^*∗*^
Pure error	13.95	4	3.49		
Total error	1427.37	12			

^*∗*^Significance.

## Data Availability

The data used to support the findings of this study are included within the article.
